# Depletion of membrane cholesterol compromised caspase-8 imparts in autophagy induction and inhibition of cell migration in cancer cells

**DOI:** 10.1186/s12935-018-0520-4

**Published:** 2018-02-20

**Authors:** Mukesh Kumar, Karuna Irungbam, Meena Kataria

**Affiliations:** Indian Veterinary Research Institute, Bareilly, India

**Keywords:** Cholesterol depletion, Caspases, Cell migration, Autophagy

## Abstract

**Background:**

Cholesterol in lipid raft plays crucial role on cancer cell survival during metastasis of cancer cells. Cancer cells are reported to enrich cholesterol in lipid raft which make them more susceptible to cell death after cholesterol depletion than normal cells. Methyl-β-cyclodextrin (MβCD), an amphipathic polysaccharide known to deplete the membrane cholesterol, induces cell death selectively in cancer cells. Present work was designed to identify the major form of programmed cell death in membrane cholesterol depleted cancer cells (MDA-MB 231 and 4T1) and its impact on migration efficiency of cancer cells.

**Methods:**

Membrane cholesterol alteration and morphological changes in 4T1 and MDA-MB 231 cancer cells by MβCD were measured by fluorescent microscopy. Cell death and cell proliferation were observed by PI, AO/EB and MTT assay respectively. Programme cell death was confirmed by flow cytometer. Caspase activation was assessed by MTT and PI after treatments with Z-VAD [OME]-FMK, mitomycin c and cycloheximide. Necroptosis, autophagy, pyroptosis and paraptosis were examined by cell proliferation assay and flow cytometry. Relative quantitation of mRNA of caspase-8, necroptosis and autophagy genes were performed. Migration efficiency of cancer cells were determined by wound healing assay.

**Results:**

We found caspase independent cell death in cholesterol depleted MDA-MB 231 cells which was reduced by (3-MA) an autophagy inhibitor. Membrane cholesterol depletion neither induces necroptosis, paraptosis nor pyroptosis in MDA-MB 231 cells. Subsequent activation of caspase-8 after co-incubation of mitomycin c and cycloheximide separately, restored the cell viability in cholesterol depleted MDA-MB 231 cells. Down regulation of caspase-8 mRNA in cholesterol depleted cancer cells ensures that caspase-8 indirectly promotes the induction of autophagy. In another experiment we have demonstrated that membrane cholesterol depletion reduces the migration efficiency in cancer cells.

**Conclusion:**

Together our experimental data suggests that membrane cholesterol is the crucial for the recruitment and activation of caspase-8 as well as its non-apoptotic functions in cancer cells. Enriched cholesterol in lipid raft of cancer cells may be regulating the cross talk between caspase-8 and autophagy machineries to promote their survival and migration. Therefore it can be explored to understand and address the issues of chemotherapeutic and drugs resistance.

**Electronic supplementary material:**

The online version of this article (10.1186/s12935-018-0520-4) contains supplementary material, which is available to authorized users.

## Background

Cholesterol is an essential structural and functional component of cells, maintains the cellular integrity and precursors for bile acid, steroid hormones and other signaling molecules [1, 2]. Most of the cholesterol concentrates in the lipid raft of plasma membrane after transportation from endoplasmic reticulum, regulate cellular proliferation, differentiation and survival [3]. Earlier reports suggested that accumulation of cholesterol in solid tumors enhances the angiogenesis and aggravate tumor formation, which is now considered as the hallmark for aggressiveness of cancer [4, 5]. Enrichment of cholesterol in the lipid raft in cancer cells make them more sensitive to cholesterol depletion and induces anoikis like cell death [6]. It shows the cholesterol in lipid raft having immense role in cancer cells survival and its progression [7]. Cholesterol enrichment in lipid raft requires for the recruitment and activation of caspase-8 and FADD in death inducing signaling complex (DISC) which execute apoptotic and non-apoptotic functions [8, 9]. Cholesterol and its metabolites are associated with initiation of different kind of programmed cell death. In recent years there are some reports suggesting induction of apoptosis and autophagy as well as anoikis like cell death mechanism in cholesterol depleted cells [10, 11]. Cholesterol depletion via cholesterol oxidase as well as cholesterol metabolites (such as 24[OH] cholesterol) was reported for induction of apoptosis and necroptosis [12, 13]. Caspase-3 activation is known for its dubiousness and can also be the results of caspase-independent cell death in cholesterol depleted cells especially due to reverse activation of caspase-8 without involvement of MOMP or receptor related signals from outside of cells [14]. Lipid raft is reported as reservoir of death inducing signaling complex where caspase-8 and FADD migrated after death receptor ligand binding. Disruption of lipid raft inhibits the migration and activation of caspase-8 [15] as well as FADD which can create opportunity for caspase independent cell death [16].

There are four major forms of caspase independent programmed cell death—autophagy, necroptosis, paraptosis and pytoptosis which are directly or indirectly associated with cholesterol metabolism [17–19]. Caspase-8 is the key regulator of many caspase independent cell deaths as well as executes many non-apoptotic functions which can be advantageous for the cancer cell’s progression [20–22]. Our initial observations on cholesterol depleted MDA-MB 231 cells was with no response to pan caspase inhibitor (Z-VAD [OME]-FMK) but a sign of programmed cell death. This inspired us to examine extensively all the major forms of programmed cell death, involvement of caspases-8 and its impact on the migration efficiency of cancer cells.

## Materials and methods

### Material

Methyl-β-cyclodextrin (Sigma, C4555-5G) soluble cholesterol (Sigma, C4951-30MG), cycloheximide (Sigma, C6255), necrostatin-1 (MerckMillipore, 480065), Z-VAD [OME]-FMK (Cayman, 187389-52-2), 3-methyl adenine (Cayman, 5142-23-4), Cholesterol assay kit (Abcam, ab133116), MTT (MPBio, 102227), Alexa Fluor^®^ 488 Annexin V/Dead Cell Apoptosis Kit (Life technology), Mitomycin C (Cayman, 1-800-364-9897); RNA isolation kit (Nucleopore, NP-84105), Revert H Minus first strand cDNA synthesis Kit (Thermo scientific, K1631).

### Cell line and cell culture

MDA-MB-231 cell line (NCCS, Pune), BALB/c 3T3 cell line (NCCS, Pune), 4T1 cell line (Kind gift from division of Animal Biotechnology, IVRI), MCF-7 cell line (NCCS, Pune), Vero cell line (NCCS, Pune), Leibovitz-15 Media (MPBIO, 1251054), DMEM (Himedia, AL151A-500 ml), RPMI-1640 (Himedia, AL162A-500 ml). All cells were maintained in 10% FBS along with penicillin (100 U/ml) and streptomycin (100 µg/ml) at 37 °C in CO_2_ incubator. MDA-MB 231 cells in Leibovitz-15 media was separately maintained.

### Cell treatment

Chemical agents were freshly prepared in recommended media and filtered with sterile syringe filter. All cells were treated with MβCD and other target agents in indicated concentration as per the protocol given for each assay.

### Qualitative estimation of membrane cholesterol

4T1 and MDA-MB 231 cells at 15000 cells/well were seeded in 96 well plate and incubated for overnight. Cells were exposed to 5 mM MβCD for 1 h in serum free media and subsequently by 1 mM soluble cholesterol (complex of MβCD and cholesterol). Cells were stained with filipin and images were collected by fluorescent microscopy [23]. Qualitative quantification of fluorescent intensity was measured and analyzed by Imagej software. Corrected total cell fluorescence (CTCF) was calculated using following formula: Integrated density − [mean area of sample × mean of background fluorescence]. This CTCF was converted into percentage against controls [24].

### Cell viability assay

MDA-MB 231, 4T1, BALB/c 3T3, and Vero cells were seeded at 15,000 cells per well in 96-well microplate and after overnight incubation treated with various concentration of MβCD in serum free media for indicated period. Cytotoxicity was determined by the MTT assay, propidium iodide (PI), acridin orange (AO) and ethidium bromide (EB). Mitochondrial membrane potential was assessed by JC-1 dye as per the manufacturer’s protocol.

### Assessment of programmed cell death

MDA-MB 231 and MCF-7 cells were seeded at 0.5 × 10^5^ cells per well in 24 well plate and incubated for overnight. Next day, treated with 5 mM MβCD for 2, 4 and 6 h. Annexin-V binding assay was performed as per the manufacturer’s protocol. MDA-MB 231 cells and Vero cells were seeded at 3 × 10^5^ cells per well in six well plate and next day treated with and without pan caspase inhibitor, Z-VAD [OME]-FMK along with the 5 mM MβCD and incubated for 2, 4, 6, 12 and 24 h. Cell viability was measured by MTT, XTT and PI [25]. For Necroptosis assessment MDA-MB 231 cells were treated with Z-VAD [OME]-FMK and 5 mM MβCD in the presence and absence of necrostatin-1 for 6 h [25]. Cell death was analyzed by MTT. Assessment of autophagy was performed by incubation of MDA-MB 231 cells with 5 mM MβCD in the presence and absence of autophagy inhibitor 3-methyl adenine (3-MA) for 6 h [26]. Cell viability was estimated by PI and MTT assay. Pyroptosis assessment was performed by exposing the Z-VAD [OME]-FMK pretreated MDA-MB 231 cells to the 5 mM MβCD in the presence and absence of glycine, a pyroptosis inhibitor [27]. Cytotoxicity was quantified by MTT assay.

### Determination of the role of caspases in autophagy

MDA-MB 231 cells were seeded at 0.3 × 10^6^ cells in six well plate and next day cells were treated with 5 mM MβCD in the presence and absence of mitomycin c, a pan caspases activator [28] and cell death was assessed by JC-1 membrane potential assay and PI. In another experiment MDA-MB-231 cells were incubated with 5 mM MβCD and in presence and absence of cycloheximide (CHX) @ 50 µg/ml, a caspase-8 activator for 6 h [29]. Cell viability was measured by PI.

### Real time polymerase chain reaction

MDA-MB 231 cells were exposed to the 5 mM MβCD for 4 h, after incubation total RNA was isolated by the RNA-isolation kit as per the manufacturer’s protocol. RNA integrity was checked in 1.2% agarose gel and c-DNA synthesis was done using Revert Aid H minus First Strand cDNA synthesis kit as per the manufacturer’s protocol. Primers for caspase-8, LC-1, Beclin-1, PI3KclassIII, ATG-3, RIPK-1, Cyclophylin-A, cFLIP and PUM-1 mRNA were designed (Table 1). PUM-1 used as reference mRNA for normalization. Relative quantitation of mRNA of caspase-8, PI3KClassIII, Beclin-1, LC-1 (MAP1LC3A), ATG-3, FLIP-c, Cyclophilin-A and RIPK-1 were performed in Step One Real time PCR (Applied Bio system).

**Table 1:** Details of primers used in experiment

mRNAs	NCBI gene accession	Primers	Sequences (5′–3′)	Tm	Product size (bp)
CASPASE-8	XM_005246891.4	Forward	GTTGTGTGGGGTAATGACAATCT	58.92	183
		Reverse	CCATTCCTGTCCCTAATGCTG	58.42	
MAP1LC3A	XM_011529085.2	Forward	TCCTGAACTGAGCTGCCTCTA	60.27	131
		Reverse	CACCCAGAGGGACAACCCTA	60.55	
BECN-1	NM_001313998.1	Forward	GGTTGAGAAAGGCGAGACACG	61.52	133
		Reverse	TGTCCACTGCTCCTCAGAGT	60.18	
RIPK-1	NM_001317061.1	Forward	TGGCAGGAAAGAAGGCCC	59.56	144
		REVERSE	CCTAGGCTGCCCATGGTGTT	62.22	
CFLAR	XM_017005196.1	Forward	GCAGCCTCTTGGAGGTGGAT	61.92	116
		Reverse	CGCAGTACACAGGCTCCAGA	61.88	
ATG-3	M_001278712.1	Forward	CGAGTGAAGCAAAGCGAGGA	60.67	128
		Reverse	GACCGGACCCAGCTGTCA	61.31	
Cyclophylin-A	M_001300981.1	Forward	CTCGAATAAGTTTGACTTGTGTTT	56.17	165
		Reverse	CTAGGCATGGGAGGGAACA	58.38	
PUM-1	NM_014676.2	Forward	AGCCCAATAACAACCTGGCA	59.89	144
		Reverse	CGCCGAGAGAACTGCTACTT	59.83	
PIK3C3	XM_011526031.2	Forward	CCTGGAAGACCCAATGTTGAAG	59.18	236
		Reverse	CGGGACCATACACATCCCA	58.78	

### Cell migration assay

4T1 and MDA-MB 231 cells were seeded at 1 × 10^6^ cells per well into six well plate. Cells were washed with serum free media and treated with mitomycin c (10 µg/ml for 30 min). After incubation cells were again washed thrice with serum free media and 5 mM MβCD was given to the cells for 1 h. Scratch was made by 200 µl micro tip, cell debris was removed and cells were kept in fresh serum free media for next 24 h [30]. Multiple images of created wound were taken in subsequent interval and analyzed by Imagej analyzer.

### Statistical analysis

Statistical analysis was performed by HPSS 11.4 free online available software. Data are presented as mean ± sde of mean at least two independent experiments. One-way anova post hock Tukey’s and Dunnett’s was used for statistical significance in multiple comparisons. Paired-t test was used to compare the lesser than three groups.

## Results

### Membrane cholesterol depletion by MβCD changes the cellular morphology, can be restored after subsequent reloading of cholesterol

**Fig. 1:**
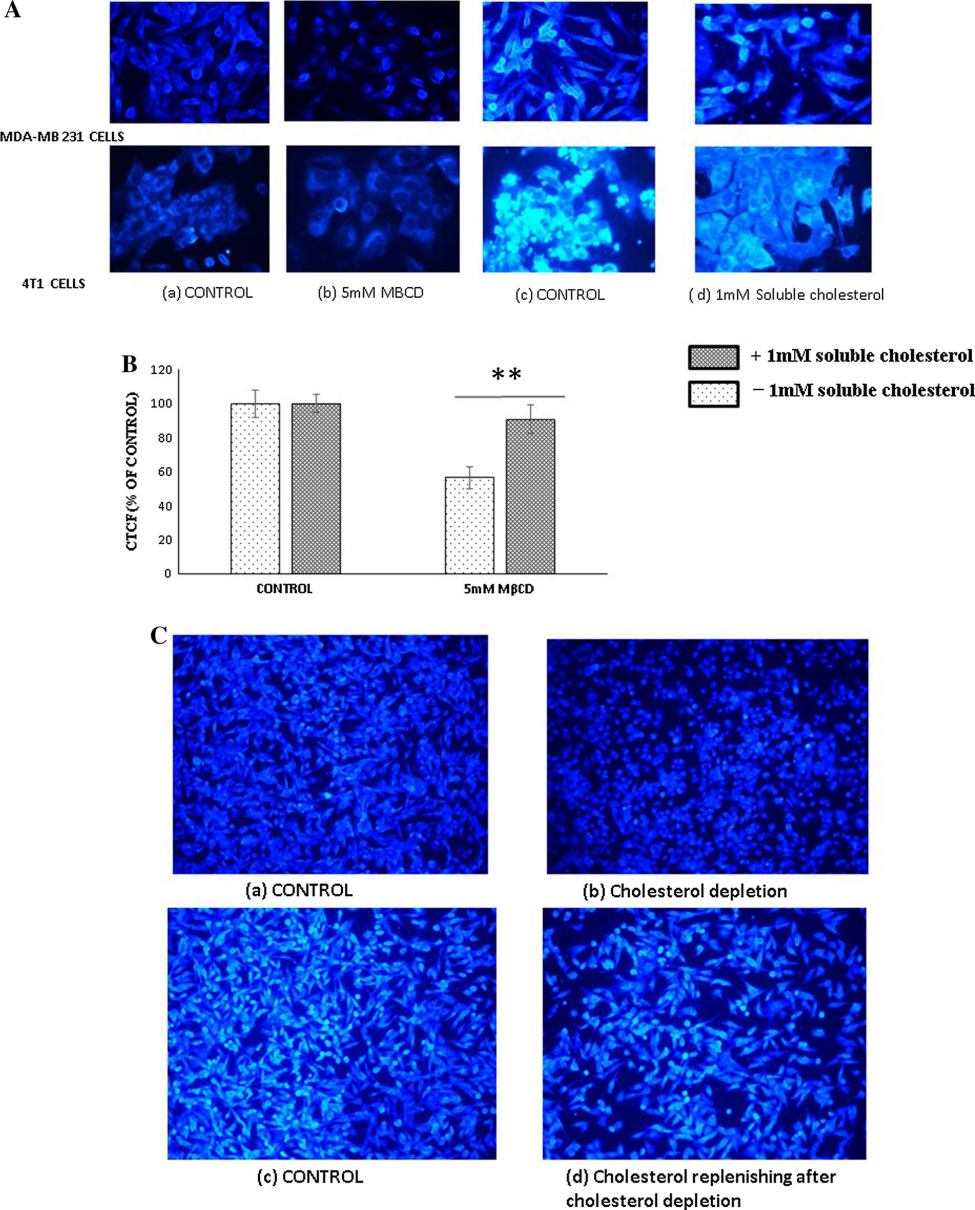
Qualitative estimation of membrane cholesterol. **A** MDA-MB 231 and 4T1 cells were treated with (**b**, **d**) and without (**a**, **c**) 5 mM MβCD for 1 h and subsequently replenished by 1 mM soluble cholesterol for next 1 h (**b**, **d**). After incubation cells were stained with Filipin III and viewed under at 20× Nikon fluorescent microscope (Additional file 1: Figure S1). **B** Fluorescent intensity was measured and CTCF (corrected total cell fluorescence) calculated by ImageJ analyzer (NIH). Statistical analysis: One way anova, post hock test Tukey. *P < 0.05, **P < 0.01, **P < 0.001, *N.S.* not significant. **C** Effect of membrane cholesterol manipulation in morphology of cells. MDA-MB 231 cells were incubated with serum free media (**a**, **c**) and 5 mM MβCD (**b**, **d**) for 1 h and subsequently with 1 mM soluble cholesterol (**c**, **d**)

In our lab, we first confirmed the potency of MβCD depleting the cholesterol from cell membrane. To examine, 4T1 and MDA-MB 231 cancer cells were incubated with 5 mM MβCD for 1 h and subsequently with 1 mM soluble cholesterol (combination of MβCD and cholesterol) in serum free media for next 1 h. Results shows that 1 h treatment of 5 mM MβCD is sufficient to deplete the cholesterol from 4T1 and MDA-MB 231 cells. Further incubation with 1 mM soluble cholesterol effectively reload the cholesterol in 4T1 and MDA-MB 231cells (Fig. 1A, B). We have also observed significant changes in the morphology of cholesterol depleted cells and subsequent reloading of cholesterol could restore the morphological changes observed in cholesterol depleted cells (Fig. 1C).

### Cholesterol depletion compromise the viability of various cell lines irrespective of species and type

Earlier reports suggested that cholesterol depletion disrupt the membrane raft which induces cell death. We first confirmed cell death after cholesterol depletion by various concentration of MβCD. Our results shows significant cell death after treatment with 5 mM MβCD in 4T1, and MDA-MB-231 cell line (Additional file 1: Figure S1a–d). Comparative study of various concentration of MβCD illustrate no significant difference among the cancerous (4T1, MDA-MB231) and non-cancerous (BALB/cc3T3) cell line (Additional file 2: Figure S2a). Certain types of cancer cells are more susceptible to death during cholesterol depletion due to enriched cholesterol in their membrane lipid raft. To explore this phenomenon, we exposed 5 mM MβCD to cancerous cell line (MDA-MB 231, 4T1 cells) and non-cancerous (MDCK, VERO, BALB’C 3T3) but could not observe any significant difference in their viability after cholesterol depletion (Additional file 2: Figure S2b).

### Pattern of cell death induced by cholesterol depletion

#### Cholesterol depletion induces programmed cell death

**Fig. 2:**
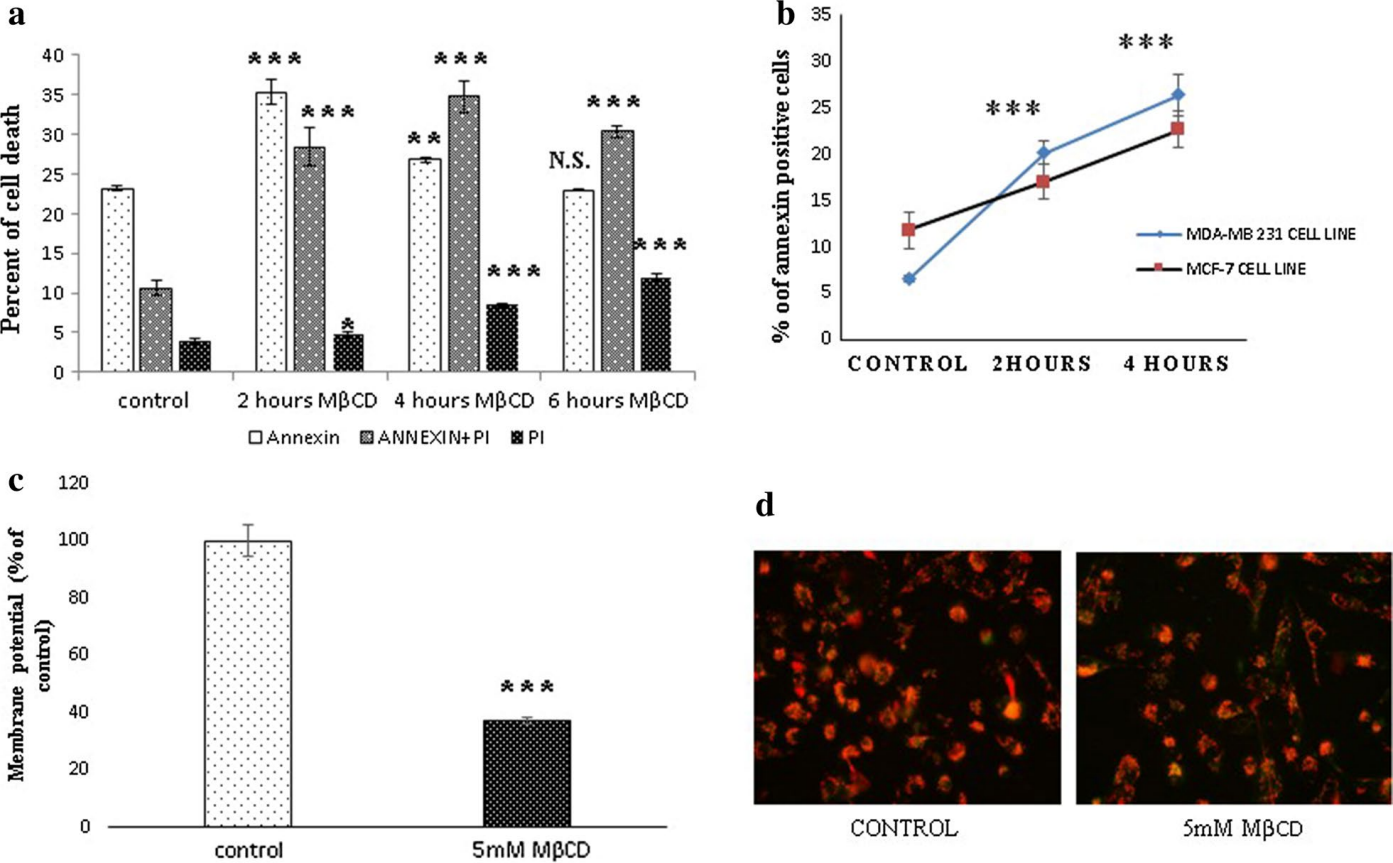
Cholesterol depletion induces programmed cell death. **a** MDA-MB 231 cells were treated with 5 mMMβCD for 2, 4 and 8 h. Cells were stained with Annexin V and PI. Exhibition of phosphatidylserine (PS) and cell death was measured by flow cytometer. **b** Phosphatidylserine exhibition by MDA-MB 231 and MCF-7 Cell line after 5 mM MβCD exposure for 2 and 4 h. **c**, **d** Flow cytometer and fluorescence microscopy analysis of membrane potential assay by JC-1 staining after 4 h exposure with 5 mM MβCD. Statistical analysis: One way anova, post hock test Dunnett’s test, *P < 0.05, **P < 0.01, **P < 0.001, *N.S.* not significant

Exposure of phosphatidylserine (PS) on outer leaflet of the cell membrane is the cardinal feature during all kind of programmed cell death. Before understanding the specific type of programmed cell death followed by cholesterol depletion, we initially confirmed the induction of programmed cell death by estimating the annexin V^+^ cell population. MDA-MB 231 cells and MCF-7 cells were treated with 5 mM MβCD for 2, 4 and 8 h. The cell viability and presence of PS was confirmed by FITC conjugated annexin V and PI. Our results shows majority of annexin^+^/PI^−^ cells within 4 h but subsequent exposure for longer duration increased the annexin^+^/PI^+^ positive cells and PI^+^ positive cells (Fig. 2a, b). Mitochondria are the signaling platform for cell death and survival mechanism. Mitochondrial depolarization is shown by all form of programmed cell death and hallmark for the apoptosis. To assess the health of mitochondria after cholesterol depletion, MDA-MB 231 cells were treated with 5 mM MβCD for 4 h and mitochondrial membrane potential was measured through JC1 dye by flow cytometer and fluorescent microscopy (Fig. 2c, d). Results indicate that mitochondrial health was compromised in cholesterol depleted cells compared to control. Hence the results from annexin-V binding and membrane potential assay together revealed induction of programmed cell death in cholesterol depleted MDA-MB 231 and MCF-7 cells.

#### Caspase independent cell death

**Fig. 3:**
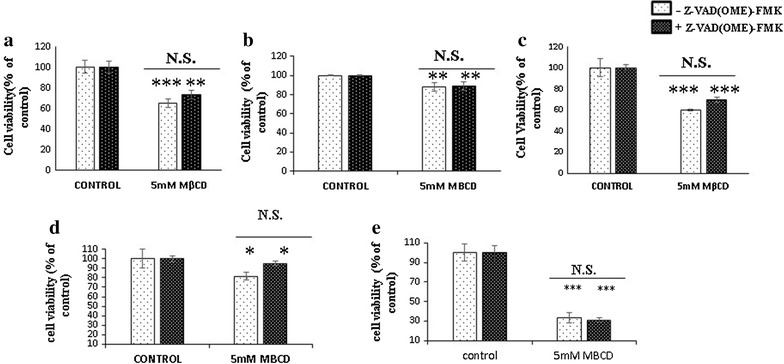
Assessment of caspase inhibition. MDA-MB 231 cells were incubated with 5 mM MβCD in the presence and absence of Z-VAD [OME]-FMK (20 µM) for **a** 2 h, **b** 4 h, **c** 6 h, **d** 12 h and **e** 24 h. Cell viability was measured by MTT (**a**–**d**) and XTT (**e**). Statistical analysis: One way anova, post hock test Tukey. *P < 0.05, **P < 0.01, **P < 0.001, *N.S.* not significant

Caspases are the major driver of apoptotic and non-apoptotic functions and mostly activated by extrinsic factors (such as TNFᾳ, infectious agents, and certain ligands of cell death receptors) and DNA damages respectively. In our experiment we used Z-VAD [OME]-FMK, a pan caspase inhibitor to know the caspases activation during cell death after cholesterol depletion. During incubation [2, 4, 6, 12, and 24 h], our results shows interestingly pan caspase inhibitor (Z-VAD [OME]-FMK) was not able to restrict cell death induced after cholesterol depletion (Fig. 3a–e). We found same results in 4T1 and Vero cell line (Additional file 3: Figure S3a–d). Hence our results showed that caspases are not directly involved in cell death induced by cholesterol depletion.

#### Cholesterol depletion neither induces necroptosis nor pyroptosis

**Fig. 4:**
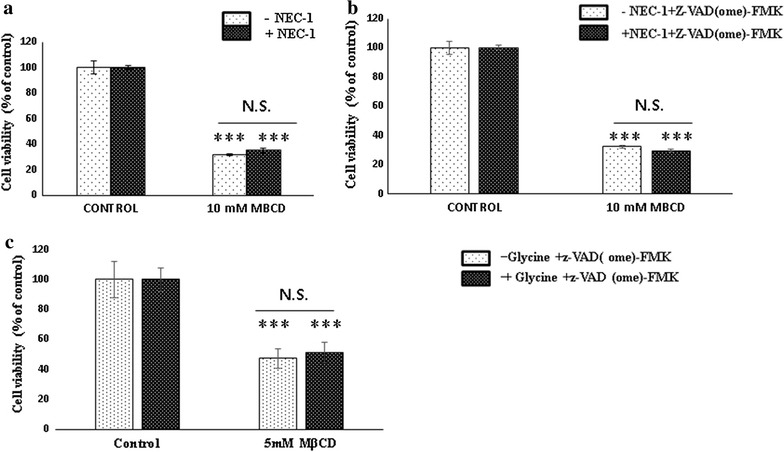
Assessment of role of necroptosis and pyroptosis. MDA-MB 231 cells were incubated with 5 mM MβCD in the presence and absence of necrostatin-1 (40 µg/ml) (**a**), Z-VAD [OME]-FMK (20 µg/ml) and necrostain-1 (40 µg/ml) (**b**) and 5 mM glycine along with Z-VAD [OME]-FMK (20 µg/ml (**c**) for 6 h. Cell proliferation was measured by MTT. Statistical analysis: One way anova, post hock test Tukey. *P < 0.05, **P < 0.01, **P < 0.001, *N.S.* not significant

Caspase independent cell death mostly predominant by autophagy, necroptosis or pyroptosis. Recently 24(S)-hydroxycholesterol was reported to induce the apoptosis and necroptosis. Necrostatin-1 inhibit the receptor interacting protein kinase-1 (RIPK-1), the key regulator of necroptosis which mostly activated during down regulation of caspase-8. To identify the induction of necroptosis in cholesterol depleted cells, MDA-MB 231 cells were co-incubated with 5 mM MβCD in the presence and absence of necrostatin-1, where we did not get any significant difference in cell death in presence of necrostatin-1 (Fig. 4a). As Z-VAD [OME]-FMK is known to promote the activation of RIPK-1 by suppressing caspase-8. We used this property to evaluate the necroptosis in cholesterol depleted cells. MDA-MB 231 cells were incubated with 5 mM MβCD in the presence of pan caspase inhibitor (Z-VAD [OME]-FMK). Results shows necrostatin-1 was not able to restore cell viability (Fig. 4b). Pyroptosis mostly occurs during the inflammation or intracellular infectious agents which mostly mediate through caspase-1 activation. Pan caspase inhibitor Z-VAD [OME]-FMK known to inhibit the caspase-1 as well as glycine, inhibits nonspecifically the membrane ion flux requires to initiates the pyroptosis. In our experiment MDA-MB 231 cells were incubated with 5 mM MβCD in presence of Z-VAD [OME]-FMK (20 µg/ml) and 5 mM glycine subsequently. It could not restore the cell viability (Fig. 4c). These results suggest that cell death induced by cholesterol depletion is neither associated with necroptosis nor pyroptosis.

#### Cholesterol depletion promotes autophagy

**Fig. 5:**
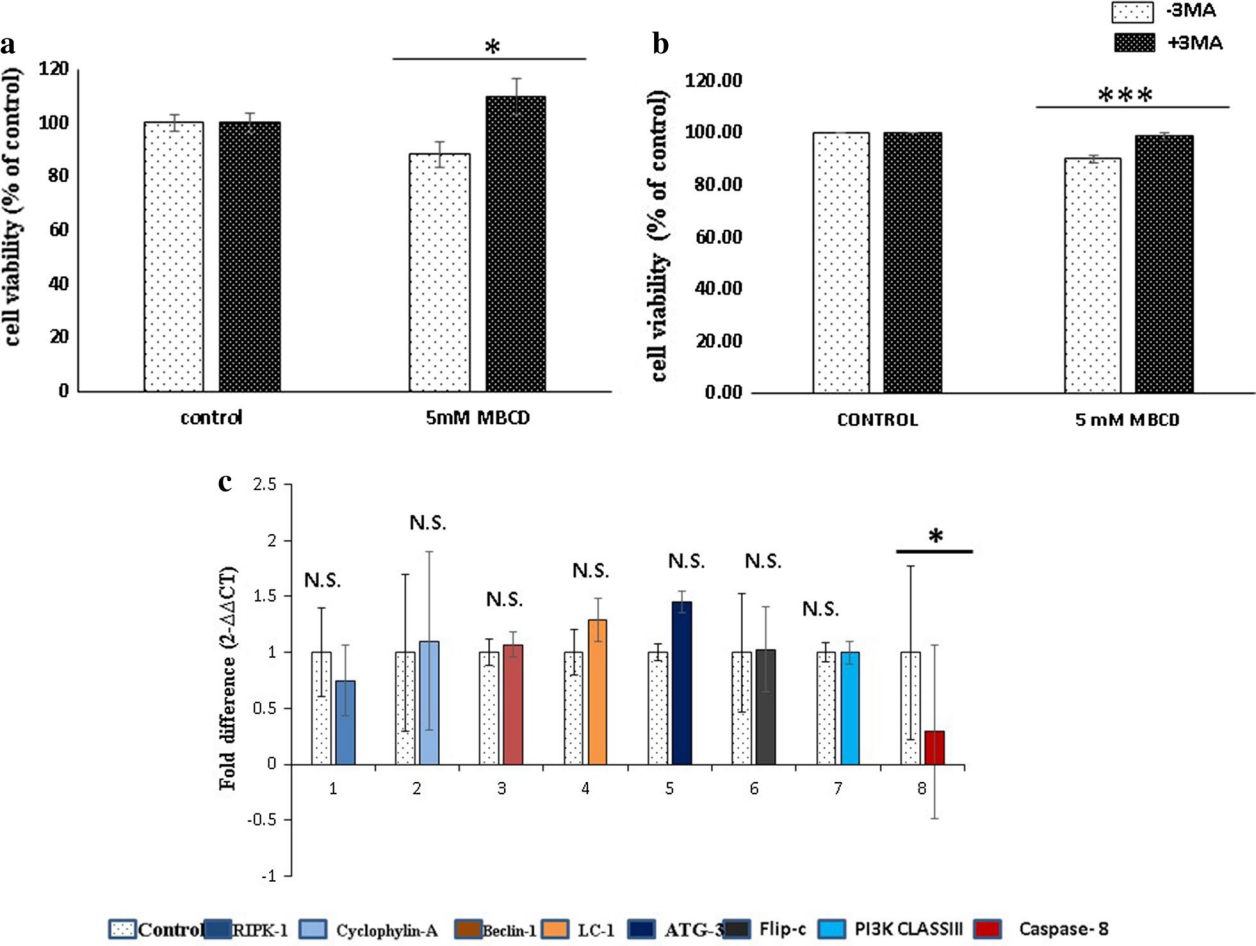
Cholesterol depletion induces autophagy. MDA-MB 231 cells were incubated with 5 mM MβCD in the presence of 3-methyl adenine (3-MA) for 6 h. Cell viability was measured by MTT and flow cytometer (**a**, **b**). Statistical analysis: One way anova, post hock test Tukey. *P < 0.05, **P < 0.01, **P < 0.001, *N.S.* not significant. **c** Relative mRNA expression of autophagy, necroptosis and apoptosis genes in MDA-MB-231 cell line. MDA-MB 231 cells were treated with 5 mM MβCD for 4 h in serum free media, total RNA was isolated, cDNA synthesized and relative quantitation was performed in applied bio system step one real time PCR system. Results are presented as mean ± S.E. of six independent experiments, each experiment performed in triplicates. Statistical analysis: paired t test, *P < 0.05, **P < 0.01, ***P < 0.001, *N.S.* not significant

Autophagy is most predominant form of caspase independent cell death which not only recycles the cellular component but also regulate the life span of cells. 3-Methyl adenine (3-MA) binds to PI3KclassIII, a crucial member of Beclin-1–PI3KclassIII complex, key center for autophagy induction. We explored the property of 3-MA to inhibit PI3K class III to unravel the cell death induced by cholesterol depletion. MDA-MB231 cells were co-incubated with 5 mM MβCD and 3-MA for 6 h. Surprisingly 3-MA significantly inhibits the cell death induced by the cholesterol depletion. It indicates that autophagy induction is the major cell death mechanism after cholesterol depletion (Fig. 5a, b). We also explored the relative quantitation of mRNAs associated with autophagy and necroptosis (RIPK-1, Flipc, Beclin-1, ATG-3, PI3KclassIII, Cyclophylin A and LC-1) But contrastingly we have not observed any significant differences in m-RNAs associated with autophagy and necroptosis (Fig. 5c).

#### Autophagy after cholesterol depletion can be attenuated by mitomycin c and cylohexamide

**Fig. 6:**
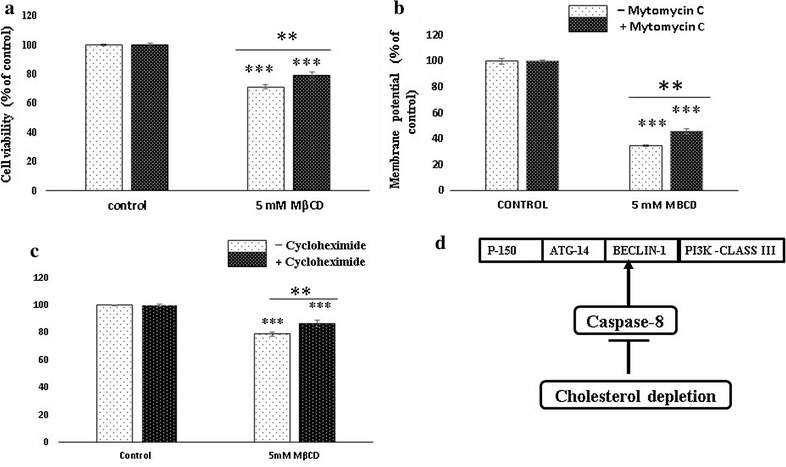
Caspase-8 activation attenuates cell death. MDA-MB 231 cells were incubated with 5 mM MβCD in the presence and absence of mitomycin c (60 µg/ml) (**a**, **b**) and cycloheximide (**c**) for 6 h. Cell viability and mitochondrial membrane potential was measured by flow cytometer. Schematic diagram of caspase-8 and promotion of autophagy in lipid raft disruption (**d**). Statistical analysis: One way anova, post hock test Tukey. *P < 0.05, **P < 0.01, **P < 0.001, *N.S.* not significant

Caspase-8 is the key regulator of caspase-independent cell death. Down regulation of caspase-8 promotes onset of autophagy and necroptosis. Mitomycin c is known to mediate the apoptosis by activation of both caspase-8 and caspase-9. We utilized the property of mitomycin c to attenuate the cell death after cholesterol depletion. MDA MB-231 cells were treated with 5 mM MβCD in the presence and absence of mitomycin c for 6 h. Cytotoxicity was measured by PI and mitochondrial membrane potential assays. MβCD treated Cells have shown significant reduction in cell death in presence of mitomycin c (Fig. 6a, b). It shows that caspase-8 down regulation plays indirect role for induction of an autophagy mediated cell death in MDA-MB 231 cells. We further confirmed it by relative quantitation of caspase-8 mRNA in which cholesterol depleted cells shown significantly 3.426 fold down regulation compared to untreated cells (Fig. 6d). Cycloheximide is the protein synthesis inhibitor activates the caspase-8 by inhibiting the c-Flip, a physiological inhibitor of caspase-8. We used this property of cycloheximide to assess the caspase-8 association with the onset of autophagy. In our experiment MDA-MB 231 cells were treated to 5 mM MβCD with or without cycloheximide (50 µg/ml) for 6 h. Results shows significant elevation in the viability of cholesterol depleted cells in presence of cycloheximide (Fig. 6c). Cycloheximide is known for inhibiting the paraptosis a kind of caspase independent programmed cell death but paraptosis could not be inhibited by 3 methyl adenine an autophagy inhibitor [31]. This suggests that cholesterol depletion is not associated with paraptosis but the autophagy promoted by caspase-8 down regulation. We further explored the association of caspases in autophagy by incubating the 3-MA pretreated MDA-MB 231 cells with 5 mM MβCD in the presence and absence of mitomycin C. Cell viability was observed by MTT. Interestingly we did not see any significant difference among them (Additional file 4: Figure S4a, b). This result suggests that mitomycin c mediated caspase activation did not restrict the cell death during autophagy inhibition.

### Cholesterol depletion reduces migration of MDA-MB 231 and 4T1 cancer cells

**Fig. 7:**
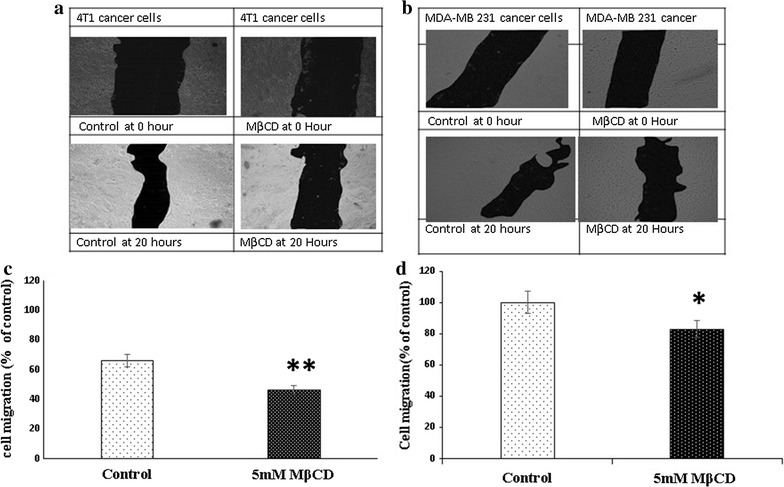
Cholesterol depletion reduces migration efficiency of cancer cells. Mitomycin c pretreated (10 µg/ml for 30 min) 4T1 (**a**) and MDA MB 231 cells (**b**) were incubated with 5 mM MβCD for 1 h and then incubated with serum scarce media for 20 h, scratch was made and multiple images were collected in different time interval and analysed by ImageJ software (**c**, **d**). Statistical analysis: Paired T test. *P < 0.05, **P < 0.01, **P < 0.001, *N.S.* not significant

Actin polymerization is the major driver for cell migration regulated by the integrin and Focal adhesion complex. Lipid raft disruption leads actin-cytoskeleton rearrangement which alters the machineries of migration. We examine the impact of cholesterol depletion in migration of MDA-MB 231 and 4T1 cancer cells. Our result showed significant down regulation of cell migration in cholesterol depleted cancer cells (Fig. 7a–d).

## Discussion

Lipid raft in plasma membrane is known as signaling platform for cellular proliferation and survival. Elevated level of cholesterol are reported in various tumors especially in chemo resistance cancer cells [32]. Raft disruption induces more cell death in certain cancer cell line comparable to normal counterpart [11]. Recent publications emphasized on the role of 24-hydroxycholesterol and cholesterol oxidase in induction of necroptosis and apoptosis [12, 13]. However apoptosis and autophagy induction after cholesterol depletion was reported earlier. They described down regulation of Bcl-xl, Akt inactivation, caspase-3 activation and accelerate conversion of LC-I to LC II after raft disruption [10]. But caspase-3 activation and autophagy induction are contrasting phenomenon. In our initial experiment on incubation of cholesterol depleted cells with pan caspase inhibitor (Z-VAD [OME]-FMK) shows no significant differences in cell death in cholesterol depleted cells. These contrasting observations motivated us to extensively explore the mechanism associated with programmed cell death, subsequently the pivotal role of caspase-8 in migration potential of cancer and non-cancer cells.

In our study we first confirmed the MβCD mediated depletion of the membrane cholesterol and its subsequent replenishment by the complex of MβCD and cholesterol in 4T1 and MDA-MB 231 cells. Depletion of cholesterol changed the morphology of MDA-MB 231 cells but subsequent enrichment restored the cellular morphology through cytoskeletal rearrangement which was consistent with the earlier report [11]. Cholesterol helps to maintain the membrane fluidity. Adhesion of cells with substrata is essentials for survival of cells and changes in morphology initiates the anoikis like cell death [33]. In our experiment cholesterol depletion was shown significant cytotoxicity from 5 mM MβCD in 4T1, BALB/c 3T3 and MDA-MB 231 cells. Although we have not observed any significant differences in cell death among the cancerous and non-cancerous cell lines with 5 mM MβCD concentration. More probably due to highly diversification in number, type and size of lipid raft among cells of different origins [34]. Hyper susceptibility of MDA-MB 231 and MCF-7 cancer cells to the cholesterol depletion compared to MCF-10A cells were shown earlier [11]. After confirming cell death in various cell lines, we next found out the existence of programmed cell death in cholesterol depleted cells. Co-incubation of MDA-MB231 and MCF-7 cells with 5 mM MβCD undermined the mitochondrial health and induces programmed cell death. Mitochondrial depolarization is the marker for the compromised mitochondria which can be assessed by JC1 dye. In our experiment we identified the expression of PS, a hallmark for early apoptosis as well as the mitochondrial depolarization within 6 h of cholesterol depletion which ensures onset of programmed cell death in MDA-MB 231 and MCF-7 cells. Caspases are bedding for both caspase dependent and independent cell death [33]. Caspase-8, caspase-9 and caspase-1 are the major driver of apoptosis and pyroptosis respectively [35]. Incubation of MDA-MB 231 cells with pan caspase inhibitor (Z-VAD [OME]-FMK) after cholesterol depletion brings no significant changes in cell death. Even relative quantitation of caspase-8 mRNA shows significant down regulation in cholesterol depleted MDA-MB 231 cells. Together our finding shows that disruption of lipid raft by MβCD follows caspase independent cell death and caspase-8 down regulation might be associated with certain kind of program cell death. Caspase-1 activation is crucial for pyroptosis and directly dependent on caspase-8 for processing [35]. Our experiment with pan-caspase (Z-VAD [OME]-FMK) and Glycine inhibitor together, nullified the existence of pyroptosis in cholesterol depleted MDA-MB 231 cells. Absence of pyroptosis indirectly supports the downregulation of caspase-8, as it requires for the activation of caspase-1.

Necroptosis, autophagy and paraptosis are the major caspase independent cell death. Caspase-8 down regulation is essential for caspase independent cell death because it cleaves the substrates necessary for autophagy and necroptosis [36]. Product of cholesterol metabolism such as 24(S)-hydroxycholesterol and 4-cholesten-3-one were found to be associated with RIPK-1 activation in neurons and ROS mediated induction of autophagy in cancer cells [32]. We were also expecting such response in cholesterol depleted cells, but our study revealed absence of necroptosis in cholesterol depleted cells. There was no significant difference in cell death when cells were co-incubated with necrostatin-1 (a necroptosis inhibitor) and 5 mM MβCD in presence and absence of pan caspase inhibitor (Z-VAD [OME]-FMK). Release of the chromatin protein high mobility group B1 (HMGB1) and Cyclophylin-A are reported as marker for late and early necrotic cell death [37]. To confirm, we performed relative quantitation of Cyclophylin-A, cFlip and RIPK-1, results shows no significant difference at mRNA level. It shows that down regulation of caspase-8 do not favor the promotion of receptor interacting protein kinase-1 (RIPK-1) but something else. Cytotoxic stimuli in compromised caspase-8 in presence of Z-VAD [OME]-FMK can activate the autophagy death in certain cell lines [33, 34]. 3-Methyl adenine (3-MA) is the inhibitor of PI3Kclass III–Beclin-1 complex, inhibit the autophagy initiation [38]. When MDA-MB 231 cells were co-incubated with 3-MA and 5 mM MβCD, cell death was significantly reduced. It indicates that Beclin-1–PI3K class III complex playing role in onset of autophagy in cholesterol chelated cells and facilitated by down regulation of caspase-8. We gained more confident when treatment of MDA-MB 231 cells with mitomycin c (60 µg/ml) and cycloheximide (50 µg/ml) restore the viability of cell death in cholesterol depleted cells. Mitomycin c is known for DNA damage, activation and processing of caspase-9 and caspase-8 whereas cycloheximide repress the cFLIP and finally stimulate the caspase-8 [28, 29]. Activation of caspase-8 by these agents may cleave Beclin-1 and ATG-3 and reduces the autophagy which culminate in more viability of cholesterol depleted cells. This finding was further confirmed when caspase-8 activation did not abrogate cell death in cholesterol depleted cells, simultaneously treated with 3-MA, a PI3Kclass III–Beclin-1 complex inhibitor. These results strongly suggested that compromised caspase-8 facilitate autophagy by promotion of Beclin-1–PI3K class III complex which can be abrogated by caspase-8 activation. Although relative quantitation of PI3K class III, Beclin-1, ATG-3 and LC1 at transcription level not revealed any significance differences. Because processing and interaction between caspases and autophagy components might be taken place at cellular or translational level [39]. Characteristics feature of paraptosis shows inhibition with cycloheximide and no response to pan caspase inhibitor and 3-MA an autophagy inhibitor [31]. However, we clearly observed both cycloheximide and 3-MA stops MβCD induced cell death. Hence our results reject the occurrence of paraptosis in cholesterol depleted cells. Cholesterol depletion might be associated to the upstream of autophagy regulator such as ULK-1 complex or mTOR complex known for nutritional sensitive, this requires further study in future. Recently it was claimed that cycloheximide inhibit the starvation induced autophagy by activating the mTORC-1 [40] but here we had also confirmed our observations with mitomycin c a potent caspase-8 activator in different combinations. However, we cannot ignore the direct or indirect (mitochondrial mediated) induction of autophagy via downregulation of mTOR1 which need investigations in future.

Cell migration is an important physiological phenomenon occurs during embryonic development, tissue organization, disease development as well as cancer progression [41]. Actin polymerization is the key player for the migration of cells which controlled by highly complexed multi nodal regulatory components [42]. Alteration in membrane cholesterol disrupt the actin cytoskeletal organization and detached the cells from substrata. We have implicated this observation to assess the migration efficiency of MDA-MB 231 cells and 4T1 cells. Apart from apoptosis, caspase-8 also involved in the regulation of cellular proliferation and cell migration [9, 43]. Cholesterol enrichment reported to promote the ᾳ6β1 integrin-mediated adhesion and assembly of the focal adhesion complex recruit caspase-8 to promote the cell migration [44]. In our experiment we noticed that cholesterol depletion significantly reduces the migration efficiency of MDA-MB 231 and 4T1 cells. Taking together our findings suggest that down regulation of caspase-8 during cholesterol depletion retard the migration efficiency of MDA-MB 231 and 4T1 cancer cells. Though we have not found significantly differences in migration efficiency between cancer (MDA-MB 213 cells) and non-cancer cell line (Vero cells) (data not included). Highly diversification in the size and shape of lipid raft in among cell lines might be the major restriction to assess the cell migration in cancer and non-cancer cell lines [34]. Moreover other study in same line shows depletion of membrane cholesterol leads down regulation of FAK and disrupt the actin cytoskeletal organization which in turn stops caspase-8 recruitment and reduce the cell migration [45]. Although we had not studied the invasiveness and epithelial mesenchymal transition in cholesterol depleted cells but similar studies suggested that MβCD mediated cholesterol depletion also reduces the cancer cell invasion and epithelial mesenchymal transition (EMT) [46–50].

## Conclusion

Conclusively our data suggests that lipid raft disruption induces caspase-independent cell death where compromised caspase-8 promotes autophagy. Neither necroptosis, pyroptosis nor paraptosis are associated with cell death after cholesterol depletion in MDA-MB 231 cells. Our preliminary data suggests that cholesterol depletion reduce the migration efficiency in MDA-MB 231 cells due to compromised caspase-8 expression and recruitment. In future there is need to explore how membrane cholesterol regulate cross talk between caspase-8 and autophagy machineries and cell migration in MDA-MB 231 cells? In future, further research in these areas can help to device novel strategies and therapeutic interventions for cancer patient.

## Additional files

**Additional file 1: Figure S1.** Cholesterol depletion induced cell death in cancer cells. MDA MB-231 Cells were incubated with 5mM MβCD at different concentration for 24 hours and cell viability was measured by MTT and PI respectively [a &b]. 4T1 Cells were treated with Methyl β Cyclodextrin at different concentration for 24 hour. Cytotoxicity was measured by MTT assay [c] and Acridine orange and ethidium bromide [AO/EB] [d]. Statistical analysis: One way anova, Post hock test Tukey. P*<0.05 P**<0.01, P**<0.001, N.S.-Not significant.

**Additional file 2: Figure S2.** Cholesterol depletion induced cell death in various cell lines. **[a]** cholesterol depletion induced cell death. MDA-MB 231, 4T1 and Balbc3T3 Cell lines were treated with different concentration of MβCD for 24 h. Cell viability was measured by MTT assay. 2[b] Vero, MDCK, 4T1, Balb/c3T3 and MDA-MB 231 cells were exposed to the 5mM MβCD for 16 h and cell death were measured by MTT assay. Statistical analysis: One way anova, post hock test Tukey. P*<0.05 P**<0.01, P**<0.001, N.S.-Not significant.

**Additional file 3: Figure S3.** Assessment of role of caspase. 4T1 Cells for 4 hour [**a**] and Vero cells[**b, c, d**] for 2, 4 and 6 hour were incubated with 5 mM MβCD in the presence and absence of Z-VAD[OME]-FMK[60 µg/ml]. Cell viability was measured by Flow cytometer [**a**], MTT [**b, c, d**]. Statistical analysis: One way anova, post hock test Tukey. P*<0.05 P**<0.01, P**<0.001, N.S.-Not significant.

**Additional file 4: Figure S4.** Role of Caspase-8 activation in cholesterol depleted cells. MDA-MB 231 cells were incubated with 5 mM MβCD and 3-Methyl adenine [3-MA] in presence and absence of mitomycin c for 6 Hours. Cell viability was measured by flow cytometer and MTT [**a**]-[**b**]. Statistical analysis: One way anova, post hock test Tukey. P*<0.05 P**<0.01, P**<0.001, N.S.-Not significant.

## Abbreviations

MβCD: methyl β cyclodextrin
PUM1: pumilio RNA binding family member 1
PIK3C3: phosphatydil Inositol 3 Kinase ClassIII
BECN1: Beclin-1
Nec-1: necrostatin-1
3MA: 3-methyl adenine
CTCF: corrected total cell fluorescence
RIPK-1: receptor interacting proteinkinase-1
c-Flipc: cellular FLICE [FADD-like IL-1β-converting enzyme]-inhibitory protein
ATG-3: autophagy related 3
PI: propidium iodide
HMGB1: protein high mobility group B1
