# Supplementary figures and images for: Depletion of membrane cholesterol compromised caspase-8 imparts in autophagy induction and inhibition of cell migration in cancer cells
Source: Cancer Cell Int. 2018 Feb 20;18:23. doi: 10.1186/s12935-018-0520-4 (PMC5819249; doi:10.1186/s12935-018-0520-4)

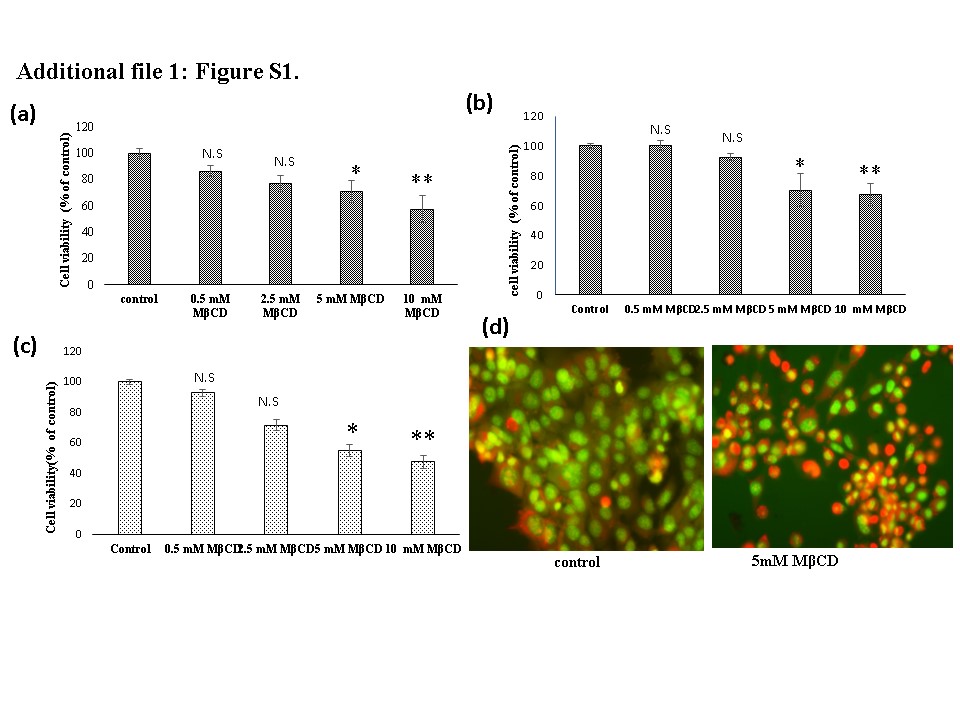

Supplement: Supplementary file 1 — Additional file 1: Figure S1. Cholesterol depletion induced cell death in cancer cells. MDA MB-231 Cells were incubated with 5mM MβCD at different concentration for 24 hours and cell viability was measured by MTT and PI respectively [a &b]. 4T1 Cells were treated with Methyl β Cyclodextrin at different concentration for 24 hour. Cytotoxicity was measured by MTT assay [c] and Acridine orange and ethidium bromide [AO/EB] [d]. Statistical analysis: One way anova, Post hock test Tukey. P*<0.05 P**<0.01, P**<0.001, N.S.-Not significant. [file 12935_2018_520_MOESM1_ESM.jpg]

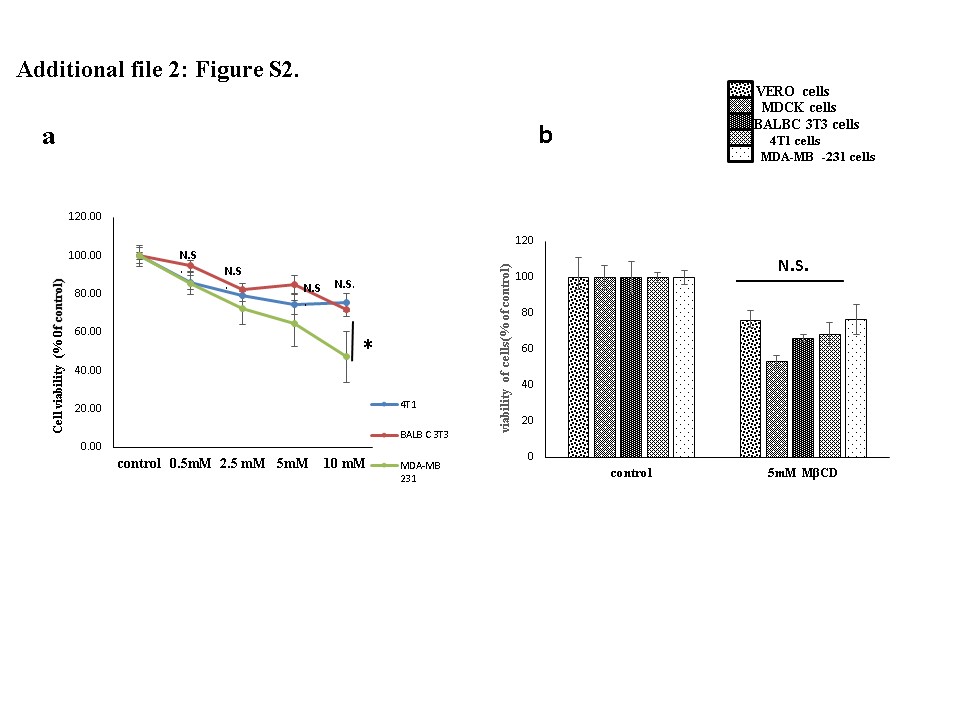

Supplement: Supplementary file 2 — Additional file 2: Figure S2. Cholesterol depletion induced cell death in various cell lines. [a] cholesterol depletion induced cell death. MDA-MB 231, 4T1 and Balbc3T3 Cell lines were treated with different concentration of MβCD for 24 h. Cell viability was measured by MTT assay. 2[b] Vero, MDCK, 4T1, Balb/c3T3 and MDA-MB 231 cells were exposed to the 5mM MβCD for 16 h and cell death were measured by MTT assay. Statistical analysis: One way anova, post hock test Tukey. P*<0.05 P**<0.01, P**<0.001, N.S.-Not significant. [file 12935_2018_520_MOESM2_ESM.jpg]

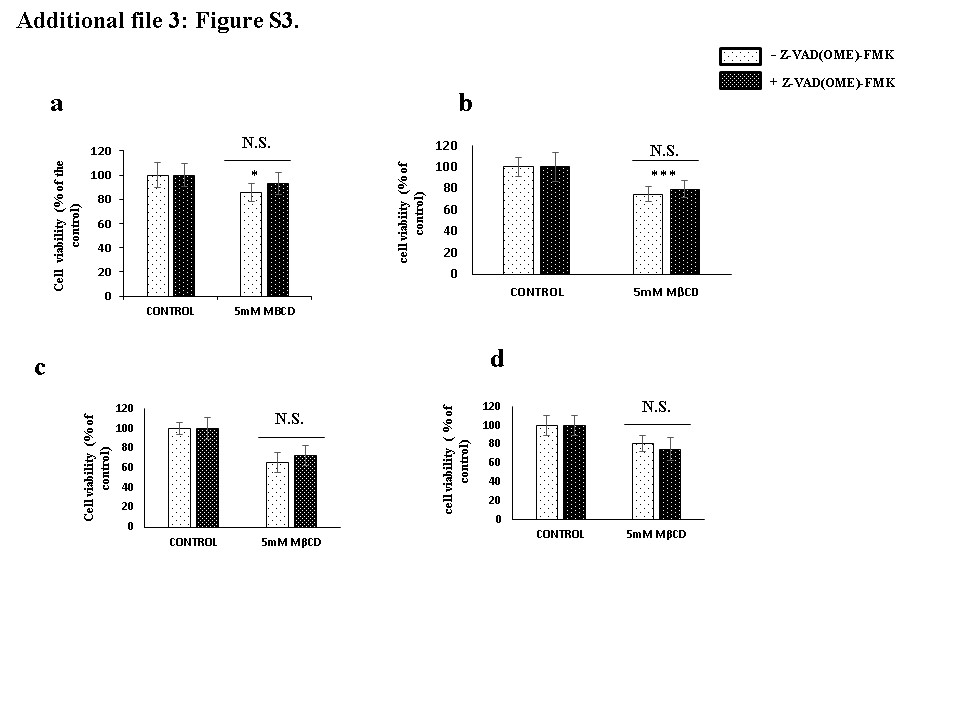

Supplement: Supplementary file 3 — Additional file 3: Figure S3. Assessment of role of caspase. 4T1 Cells for 4 hour [a] and Vero cells[b, c, d] for 2, 4 and 6 hour were incubated with 5 mM MβCD in the presence and absence of Z-VAD[OME]-FMK[60 µg/ml]. Cell viability was measured by Flow cytometer [a], MTT [b, c, d]. Statistical analysis: One way anova, post hock test Tukey. P*<0.05 P**<0.01, P**<0.001, N.S.-Not significant. [file 12935_2018_520_MOESM3_ESM.jpg]

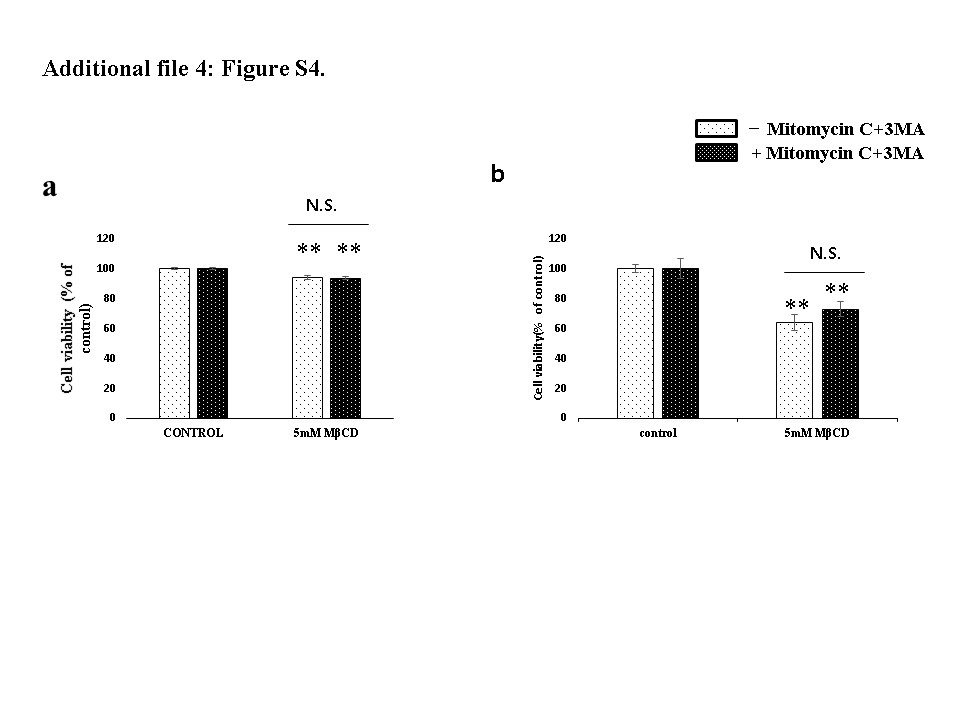

Supplement: Supplementary file 4 — Additional file 4: Figure S4. Role of Caspase-8 activation in cholesterol depleted cells. MDA-MB 231 cells were incubated with 5 mM MβCD and 3-Methyl adenine [3-MA] in presence and absence of mitomycin c for 6 Hours. Cell viability was measured by flow cytometer and MTT [a]-[b]. Statistical analysis: One way anova, post hock test Tukey. P*<0.05 P**<0.01, P**<0.001, N.S.-Not significant. [file 12935_2018_520_MOESM4_ESM.jpg]
